# Scarlatina Simplex

**Published:** 1840-01

**Authors:** 


### SCARLATINA SIMPLEX.

For several years past, different varieties of Scarlatina have prevailed, some where in the West. In one year, one town has been affected, in another a different place. In degree, it has varied from high and fatal malignancy, to extreme mildness. During the present winter in Louisville, it has assumed the latter character, presenting itself as a slight febrile affection, with moderate tumefaction of the glands and ganglia of the throat and neck, followed in several cases by œdema of the lower extremities. In some families most of the children have been, either simultaneously or successively affected. We shall be happy to receive for publication, an account of this epidemic, as it has latterly shown itself in the West. D.

